# PBP1a-Deficiency Causes Major Defects in Cell Division, Growth and Biofilm Formation by *Streptococcus mutans*

**DOI:** 10.1371/journal.pone.0124319

**Published:** 2015-04-16

**Authors:** Zezhang T. Wen, Jacob P. Bitoun, Sumei Liao

**Affiliations:** 1 Department of Comprehensive Dentistry and Biomaterials, Louisiana State University Health Sciences Center, New Orleans, LA, 70112, United States of America; 2 Center of Oral and Craniofacial Biology, Louisiana State University Health Sciences Center, New Orleans, LA, 70112, United States of America; 3 Department of Microbiology, Immunology and Parasitology, Louisiana State University Health Sciences Center, New Orleans, LA, 70112, United States of America; Centers for Disease Control & Prevention, UNITED STATES

## Abstract

*Streptococcus mutans*, a key etiological agent of human dental caries, lives almost exclusively on the tooth surface in plaque biofilms and is known for its ability to survive and respond to various environmental insults, including low pH, and antimicrobial agents from other microbes and oral care products. In this study, a penicillin-binding protein (PBP1a)-deficient mutant, strain JB467, was generated by allelic replacement mutagenesis and analyzed for the effects of such a deficiency on *S*. *mutans*’ stress tolerance response and biofilm formation. Our results so far have shown that PBP1a-deficiency in *S*. *mutans* affects growth of the deficient mutant, especially at acidic and alkaline pHs. As compared to the wild-type, UA159, the PBP1a-deficient mutant, JB467, had a reduced growth rate at pH 6.2 and did not grow at all at pH 8.2. Unlike the wild-type, the inclusion of paraquat in growth medium, especially at 2 mM or above, significantly reduced the growth rate of the mutant. Acid killing assays showed that the mutant was 15-fold more sensitive to pH 2.8 than the wild-type after 30 minutes. In a hydrogen peroxide killing assay, the mutant was 16-fold more susceptible to hydrogen peroxide (0.2%, w/v) after 90 minutes than the wild-type. Relative to the wild-type, the mutant also had an aberrant autolysis rate, indicative of compromises in cell envelope integrity. As analyzed using on 96-well plate model and spectrophotometry, biofilm formation by the mutant was decreased significantly, as compared to the wild-type. Consistently, Field Emission-SEM analysis also showed that the PBP1a-deficient mutant had limited capacity to form biofilms. TEM analysis showed that PBP1a mutant existed primarily in long rod-like cells and cells with multiple septa, as compared to the coccal wild-type. The results presented here highlight the importance of *pbp1a* in cell morphology, stress tolerance, and biofilm formation in *S*. *mutans*.

## Introduction

*Streptococcus mutans*, a key etiological agent of dental caries, lives almost exclusively on the tooth surface in biofilms known as dental plaque. *S*. *mutans* possesses multiple mechanisms to colonize the tooth surface [1–4]. *S*. *mutans* possesses at least three glucosyltransferases (Gtfs) that utilize dietary sucrose as substrate to produce high molecular weight, highly adhesive extracellular glucans (also mutans) [4–6]. The adhesive glucans function as scaffold and facilitate adherence to substrata and inter-cellular interactions and thus, biofilm accumulation via glucan-binding proteins (Gbps). Surface-associated protein P1 (also SpaP) is a high affinity adhesin that the bacterium utilizes to adhere to the tooth surface via interactions with salivary agglutinins. In addition, this bacterium also actively releases extracellular deoxyribonucleic acids (eDNA) that form nanofiber network facilitating cell-surface and cell-cell interactions [2]. *S*. *mutans* is known for its ability to survive and respond to various environmental insults, low pH and various toxic metabolites.

The cell envelope is essential in maintenance of cell shape, cell growth and cell division, and in protection against various environmental insults, including toxic metabolites and antimicrobials. The cell envelope is also directly involved in environmental signaling and bacterial cell-surface and cell-cell interactions, and thus, bacterial colonization and biofilm formation [7, 8]. The cell envelope of Gram-positive bacteria is featured with a thick layer of peptidoglycan (PG). Efficient cell division and growth requires coordinated remodeling of the PG sacculus and requires the activities of PG hydrolases, synthases, and morphogenesis proteins [9]. Penicillin-binding proteins (PBPs) function to increase resistance to β-lactams antibiotics through sequestration, although some PBPs have low-affinity for β-lactams and function in maintaining proper cell morphology. These low-affinity PBPs are thought to have evolved from horizontal gene transfer with commensal streptococci, followed by recombination events [10–14]. PBPs are grouped into two classes based on enzymatic activity towards peptidoglycan biosynthesis and/or maintenance [15–18]. Class A PBPs (PBP1a, PBP1b, and PBP2a) have both transpeptidase and glucosyltransferase activity. Class B PBPs (PBPx, PBP2x, and PBP2b) possess only transpeptidase activity. Multiple studies have been done on PBPs concerning antibiotic resistance, cell envelope biogenesis and cell division [19–24], but currently, limited information is available concerning their effects in biofilm formation, especially in *S*. *mutans* [19, 25, 26].

*S*. *mutans* possesses at least six putative PBPs homologues [27], but the function of these PBP proteins remain largely unclear. Recently, we have started to characterize the PBPs in *S*. *mutans* pathohpysiology by use functional genomics approach. Results so far have shown that *S*. *mutans* PBP1a (SMU.467c) is required for optimal growth under acidic and alkaline conditions. Also, deficiency of PBP1a causes at least a 50-fold increase in sensitivity to the redox-cycling agent paraquat. Furthermore, our results show that depletion of PBP1a leads to an increased susceptibility to both acid and hydrogen peroxide stress and major defects in biofilm formation. Lastly, PBP1a-deficiency leads to morphological changes of *S*. *mutans* cells.

## Materials and Methods

### Plasmids, bacterial strains, and growth conditions

Bacterial strains and plasmids used in this study are listed in Table 1. *S*. *mutans* strains were maintained in Brain Heart Infusion (BHI, Difco Laboratories) medium. Solid media were prepared similarly, while Bacto agar (Difco Laboratories) was added at a concentration of 1.5% (w/v). When needed, kanamycin (Kan, 1 mg mL^-1^) and/or spectinomycin (Spc, 1 mg mL^-1^) were added to the growth medium. Unless stated otherwise, cultures were grown at 37°C in an aerobic chamber containing 5% CO_2_ under static conditions. For growth characteristics, actively growing cultures were diluted 1:100 in fresh medium, and growth in culture optical density (OD_600nm_) was continuously monitored using a Bioscreen C (Oy Growth Curves AB Ltd, Finland) at 37°C with and without sterile mineral oil overlay.

**Table 1:** Bacterial strains, plasmids and primers used in this study.

Strains /Plasmid	Relevant characteristics		Sources/References
*S*. *mutans* UA159	Wild-type, serotype *c*		[27]
*S*. *mutans* JB467	Derivative of UA159, Δ*pbp1a*, Kan^r^		This study
*S*. *mutans* JB467C	JB467 carrying pDL278:*pbp1a*, Spc^r^, Kan^r^		This study
pDL278	Shuttle vector, Spc^r^		[28]
pDL278:*pbp1a*	Shuttle carrying *pbp1a*, Spc^r^		This study
Primers used	Sequence (5’ to 3’)	Sequence (5’ to 3’)	Application
467 55 and 53	55: gcttcactccagtgctctccatga	53: acagaagacctcgaattctaatggct	5’ fragment for *pbp1a* mutation
467 35 and 33	35: cagaagcgtctagtgaattctcttcatc	33: agaagcgtctagtgaattctcttcatc	3’ fragment for *pbp1a* mutation
467 seq	5: tgacagttctaaaatatggactaggt	3: aagcacactctaataccaagagtgt	*pbp1a* sequencing confirmation
467 compl	5: atggaattcaggtgaatgtgtcagctt	3: gctgaggagctcgcggcttat	Complementation

Note: Kan^r^ and Spc^r^, for kanamycin and spectinomycin resistance, respectively. Sequences underlined are restriction sites engineered for cloning.

### Construction of deficient mutant and complement strain

To construct a PBP1a-deficient mutant, a PCR-ligation-mutagenesis strategy was used as previously described to remove nucleotides 60–2066 within the coding sequence [29, 30]. Briefly, the 5’ and 3’ regions flanking *pbp1a* were amplified by PCR using Phusion high-fidelity DNA polymerase (New England Biolabs, Ipswich, MA) with gene-specific primers shown in Table 1. Following proper restriction enzyme digestions, the flanking regions were ligated to a non-polar kanamycin resistant element (*aphA* encoding aminoglycoside 3'-phosphotransferase) [31] that was digested similarly. The resulting ligation mixtures were used to directly transform *S*. *mutans* UA159 in the presence of competence stimulating peptide (CSP) [32]. An allelic replacement mutant, JB467 with *pbp1a* deficiency, was isolated on BHI-Kan plates and further confirmed using PCR and sequencing. For complementation of the JB467, the *pbp1a*-coding sequence plus its cognate promoter region were amplified by PCR, digested, and cloned into shuttle vector pDL278. After sequence confirmation, the resulting construct, pDL-*pbp1a*, was used to transform JB467, generating complement strain, JB467C.

### Biofilm analysis

*S*. *mutans* biofilms were grown in modified biofilm medium with glucose (20 mM, BMG), sucrose (10 mM, BMS), or glucose (18 mM) and sucrose (2 mM) (BMGS) as the supplemental carbohydrate sources as previously described [33, 34]. Overnight cultures were inoculated 1:100 into fresh BHI and allowed to grow until OD≅ 0.5, and then the biofilm media was inoculated by 1:100 dilutions. Biofilm mass on 96-well plates (Corning, NY) was measured spectrophotometrically after crystal violet staining [35]. For biofilm structural analysis, hydroxylapatite (HA) disks, commonly used as *in vitro* tooth model, were also used and placed vertically in fabricated orthodontic wire holders fit for 24-well plates [36], and biofilms were analyzed using scanning electron microscopy (SEM).

### Glycolytic pH drop, acid killing and hydrogen peroxide challenge assays

The impact of *pbp1a* deficiency on the ability of *S*. *mutans* to withstand acid and oxidative stress were determined by using procedures described elsewhere [29, 37, 38]. The glycolytic pH drop assay was carried out as described by Belli et al. [38]. Briefly, *S*. *mutans* strains were grown in BHI until mid-exponential phase (OD_600nm_ ≅0.5), washed twice with ice cold de-ionized water by centrifugation at 4,000 rpm at 4°C for 10 minutes, and the cells were then resuspended in 50 mM KCl and 1 mM MgCl_2_. The pH was adjusted to 7.2 with 0.1 M KOH before the addition of 50 mM glucose. The pH drop was monitored continuously for a period of 30 minutes. For acid tolerance response, middle-exponential phase culture (OD≅0.3) were properly washed and then incubated in glycine buffer, 0.1 M, pH 2.8 for periods of 30, 45 and 60 minutes [37, 39]. To evaluate superoxide stress susceptibility, paraquat (also, methyl viologen, Sigma, St. Louis, MO) was prepared freshly and added to the growth medium, BHI, and the growth impact was monitored using Bioscreen C [29]. For hydrogen peroxide killing, bacterial cells were prepared similarly as above, and then incubated in glycine buffer, 0.1 M, pH 7.0 containing 0.2% (w/v, or 58 mM final conc.) hydrogen peroxide for periods of 90 and 110 minutes as detailed elsewhere [37, 40].

### Field Emission-SEM

For field emission-scanning electron microscopic (FE-SEM) analysis, *S*. *mutans* overnight cultures were diluted 1:100 in either BMG, BMGS or BMS on HA discs placed horizontally in 24-well plates as previously described [29, 36]. Briefly, after 24 hours of growth, the HA disks were rinsed briefly with PBS, pH 7.4, and then fixed in 2.5% glutaraldehyde (Polysciences, Warrington, PA) overnight at 4°C. The fixed samples were dehydrated using increasing concentrations of ethanol, and then dried at the critical point of CO_2_ (Electron Microscopy Sciences, Hatfield, PA). A small amount of carbon was sputtered onto the samples to avoid charging in the microscope. Microscopy was performed with a Hitachi S-4800 High-resolution microscope (Tulane University, New Orleans, Louisiana).

### TEM analysis

Transmission electron microscopic (TEM) analysis was carried out similarly as described previously [29]. Briefly, *S*. *mutans* strains were grown in BHI until mid-exponential phase, harvested by centrifugation at 4,000 rpm, 4°C for 10 minutes, washed twice with PBS, and then fixed in 2% paraformaldehyde/2.5% glutaraldehyde (Polysciences, Warrington, PA) in PBS for 1 h at room temperature. Cells were washed in PBS and post-fixed in 1% osmium tetroxide (Polysciences) for 1 h. Samples were then rinsed extensively in dH_2_0 prior to en bloc staining with 1% aqueous uranyl acetate (Ted Pella Inc., Redding, CA) for 1 hour. The cells were then washed with dH_2_0 and dehydrated in a graded series of ethanol and embedded in Eponate 12 resin (Ted Pella Inc., Redding CA). Sections of 90–100 nm were prepared, stained with uranyl acetate and lead citrate, and viewed under a JEOL 1200 EX transmission electron microscope (JEOL USA Inc., Peabody, MA).

### Autolysis assay

For autolysis assay, *S*. *mutans* strains were grown in BHI overnight. Bacterial cells were harvested by centrifugation at 4,000 rpm, 4°C for 10 minutes, washed once with phosphate buffered saline (PBS, pH 7.2), and then resuspended in PBS with and without inclusion of Triton X-100 (0.2%, v/v, Sigma) and adjusted to similar OD_600nm_ [35]. The cell suspension was then incubated at 37°C, and autolysis in reduction of OD_600nm_ was monitored automatically using Bioscreen C every 30 minutes with moderate shaking (10 seconds) before measurement.

### Statistical Analysis

Quantitative data were analyzed using the paired Student’s *t*-test. A difference at *P* value equal to or less than 0.05 is considered statistically significant.

## Results

### PBP1a deficiency causes major defects in growth

**Fig 1:**
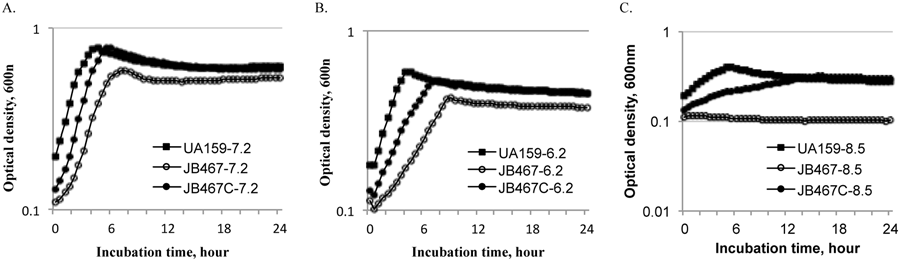
Growth study under different pHs. *S*. *mutans* wild-type (UA159, solid squares), PBP1a-deficient mutant (JB467, open circles), and the complement strain (JB467C, solid circles) were grown in BHI adjusted to pH 7.2 (A), pH 6.2 (B), and pH 8.5 (C) in Bioscreen C with sterile mineral oil overlay, and the culture optical densities were monitored every 30 minutes at 600nm. The results suggest that PBP1a-deficeincy affects growth rate and cell yield in optical density at 600 nm, especially in BHI adjusted to higher pH. The data presented here are representatives of three independent experiments.

A PBP1a-deficient *S*. *mutans* strain, JB467, was constructed by replacing nucleotides 59 to 2077 relative to the translational start site of *pbp1a* with a nonpolar kanamycin-resistance cassette by allelic replacement. When compared to the wild-type, UA159 during growth in BHI, the PBP1a-defcient mutant, JB467 had a reduced growth rate with an averaging doubling time of 135(±13) minutes, compared to 93(±3) minutes for the wild-type (*P*<0.01). Relative to the wild-type, the PBP1a mutant also had a reduced culture OD_600nm_, with the average maximal OD_600nm_ of the overnight wild-type being 0.779 vs 0.586 for the PBP1a mutant (*P*<0.005) (Fig 1A). Besides, the mutant also had higher tendency to form aggregates at the bottom of the culture tube leaving a clear growth medium broth (Data not shown). A PBP1a-complement strain, JB467C, which carries the wild-type copy of the *pbp1a* gene plus its cognate promoter region, was constructed using shuttle vector pDL278 (Table 1). Complementation in JB467C was able to restore the slow-growth phenotype of PBP1a-deficient mutant to that similar to wild-type UA159 with an averaging doubling time of 92±2.5 minutes and an OD_600nm_ maximum of 0.781 when grown under similar conditions (Fig 1A). The complementation *in trans* also restored the aggregation characteristics to the wild-type.

### PBP1a-deficient mutant is more susceptible to acid and oxidative stresses

In an effort to discern the effects of PBP1a-deficiency on the ability to tolerate stresses, the PBP1a-deficeint mutant, along with the wild-type and the complement strain, were grown in BHI adjusted to different pHs. At pH 6.2, *S*. *mutans* wild-type UA159 grew at a slower rate than that at neutral pH, as expected (Fig 1B). However, relative to the wild-type, the PBP1a-deficient mutant further reduced its growth rate, with an averaging doubling time of 232 minutes vs 115 minutes for the wild-type (*P*<0.001). Similar observations were also made with the culture density of the overnight cultures, with an averaging OD_600nm_≅0.42 for the PBP1a-deficient mutant vs OD_600nm_≅0.59 for the wild-type (*P*<0.001) (Fig 1B). The PBP1a mutant completely stopped to grow when incubated in BHI adjusted to pH 8.5 (Fig 1C). Similarly, the complementation *in trans* was able to partly restore the phenotypes to those similar to the wild-type.

**Fig 2:**
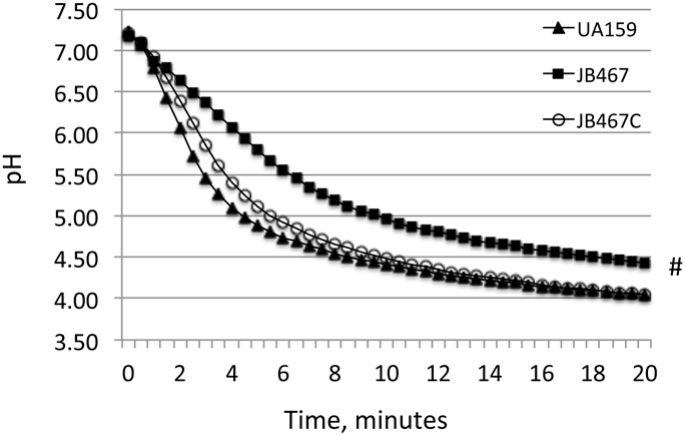
Glycolytic pH drop. Results of pH drop experiments showed that relative to *S*. *mutans* UA159 (triangles), the PBP1a-deficient mutant, JB467 (squares), had a slower pH drop and a higher resting pH after 20 minutes (#, *P*<0.01). Complementation with wild-type *pbp1a* in JB467C (open cycles) was able to restore the phenotype to the wild-type, UA159.

**Fig 3:**
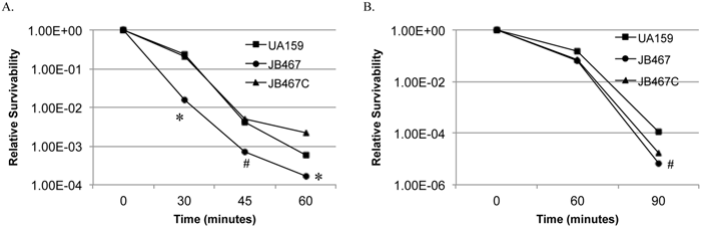
Acid (A) and hydrogen peroxide (B) killing assays. *S*. *mutans* wild-type (UA159, squares), PBP1a mutant (JB467, circles), and the complement strain (JB467C, triangles) were grown in BHI until mid-exponential phase (OD_600_nm≅0.3), washed and then subjected to acid and hydrogen peroxide killing assays by incubating the cells in buffer of pH 2.8 and buffer containing 0.2% (w/v) hydrogen peroxide, respectively, for periods of time as indicated. Results showed that PBP1a deficiency in JB467 weakens the ability of the deficient mutant to tolerate low pH (A) and hydrogen peroxide (B), when compared to the wild-type. Data presented here are representatives of three independent experiments. Significant difference is indicated by * and # at *P*<0.001 and *P*<0.01, respectively.

Reduction of growth rate during growth in BHI adjusted to lower pH indicates a reduced glycolytic activity and/or weakened acid tolerance. When analyzed using glycolytic pH drop experiments, an assay commonly used to analyze the proton permeability [38], it was shown that relative to wild-type, the rate (slope) of glucose-induced pH drop of the PBP1a-deficient mutant was significantly reduced and the resting pH of the mutant was significantly higher (*P*<0.01) (Fig 2). As expected, complementation of the mutant with the wild-type coding sequence plus its cognate promoter region in JB467C was able to restore these phenotypes to levels similar to the wild-type. When incubated in glycine buffer of pH 2.8, the PBP1a-deficient mutant, JB467, showed an increased sensitivity of more than 1-log than the parent strain, UA159 (*P*<0.001) after 30 minutes (Fig 3A). The complement strain showed a level of sensitivity that is very similar to the wild-type after 30 and 45 minutes, although had a slightly higher survivability than the parent strain after 60 minutes.

**Fig 4:**
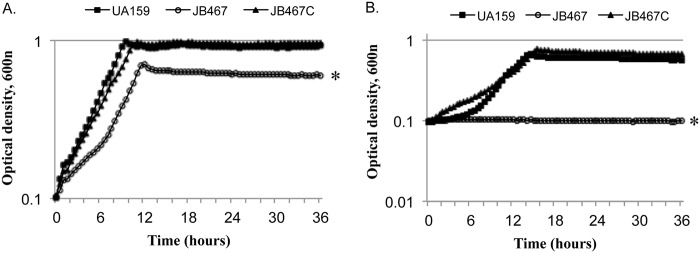
Growth study in the presence of paraquat. *S*. *mutans* wild-type (UA159, squares), PBP1a mutant (JB467, open circles), and the complement strain (JB467C, triangles) in the presence of increasing amounts of paraquat, and growth was continuously monitored using Bioscreen C with sterile mineral oil overlay. The data presented here are representative of three independent experiments, showing the increased susceptibility of the PBP1a-deficient strain, JB467, to 0.1 mM (A) and 5 mM (B) of paraquat (*P*<0.001, as indicated by *).

To assess the impact of the PBP1a-deficiency on the ability to tolerate oxidative stress, paraquat, a redox cycling agent, was included in the growth medium to generate intracellular superoxide. Relative to the wild-type, the PBP1a-deficient mutant, JB467 displayed apparent effects in both the growth rate and its cell yield in culture OD_600nm_ during growth in medium with paraquat a level as low as 0.1 mM (Fig 4A). As the concentration of paraquat was increased, the PBP1a-deficient mutant was further exacerbated (Data not shown) and almost completely eliminated growth 5 mM (Fig 4B). Complementation of *pbp1a* in *trans* in JB467C was able to restore the paraquat-sensitive growth phenotype of JB467 to the level very similar to the wild-type, UA159. When challenged with hydrogen peroxide, the PBP1a-deficient mutant was about 1-log more sensitive than the wild-type (*P*<0.001) after 90 minutes of incubation (Fig 3B). Complementation of the deficient mutant with the wild-type copy of *pbp1a* plus its cognate promoter region in multiple copy shuttle vector, pDL278, was able to only slightly increase the resistance.

### PBP1a-deficiency increases autolysis rate

**Fig 5:**
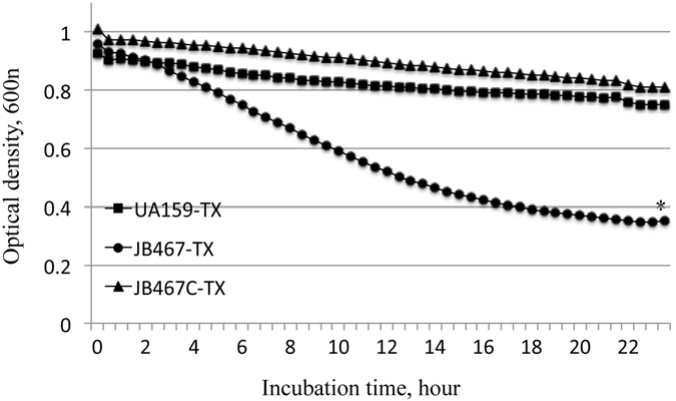
Autolysis assay. Overnight cultures of *S*. *mutans* wild-type (UA159, diamonds), PBP1a mutant (JB467, open circles), and its complement strain (JB467C, triangles) were washed and resuspended in phosphate buffered saline, pH 7.2 with and without inclusion of Triton X-100 (TX, 0.2%), and the optical density of these cells was then monitored using Bioscreen C. Relative to wild-type UA159, PBP1a mutant JB467 displayed a significantly quicker and more severe reduction of the optical density over the time of incubation (indicated by a asterisk, *P*<0.01). The graph shows results with the presence of Triton X-100 and is representative of more than three independent experiments.

When resuspended in PBS with inclusion of Triton X-100, the PBP1a-deficeint mutant, JB467 reduced its density a lot faster than the parent strain, UA159, and after 24 hours, reached a density level significantly lower than the parent strain (*P*<0.01) (Fig 5). Similar results were also observed when resuspended in PBS without Triton X-100 (Data not shown).

### PBP1a deficiency abolishes biofilm formation

**Fig 6:**
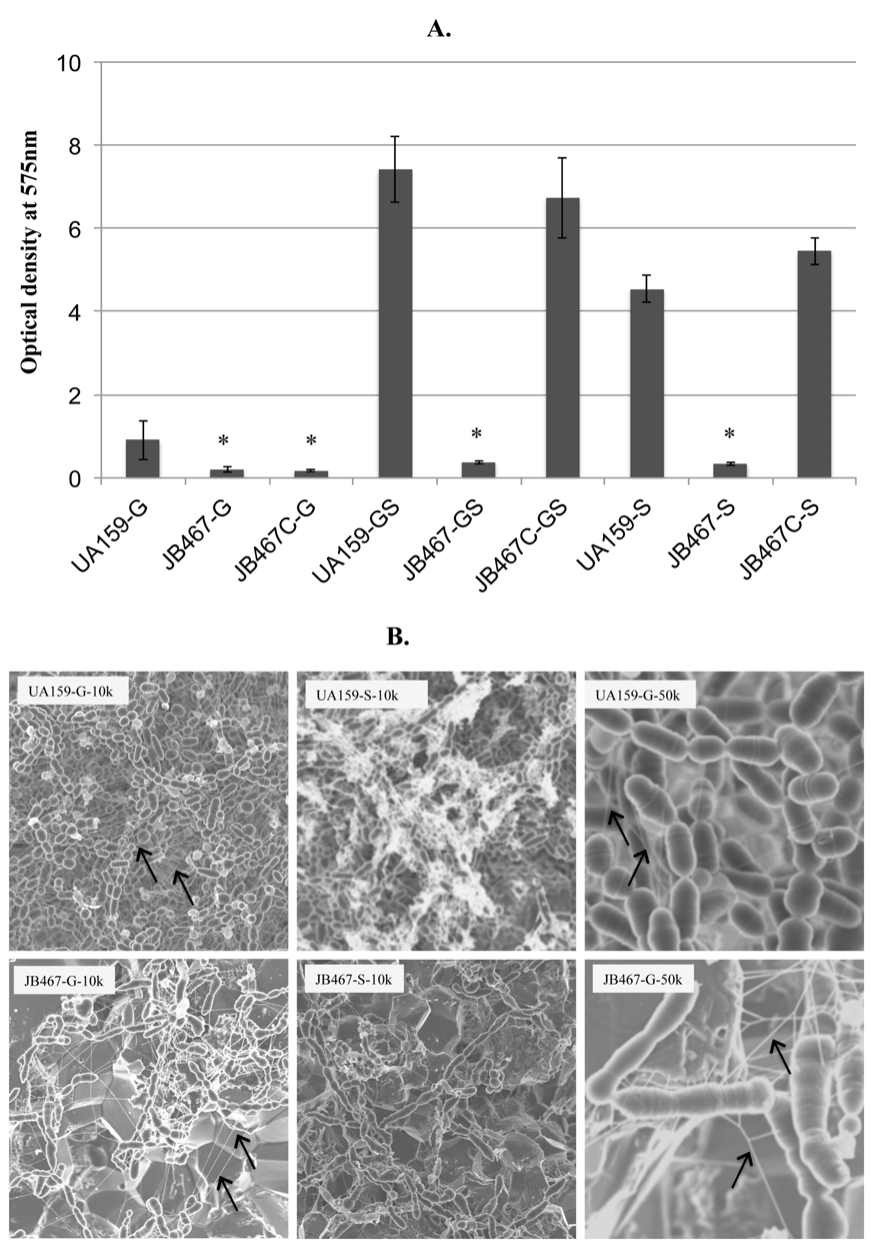
Biofilm formation assays. *S*. *mutans* wild-type (UA159), PBP1a-deficient mutant (JB467), and the complement strain (JB467C) were grown in 96-well plates or HA discs in biofilm media with glucose (G), sucrose (S), and glucose plus sucrose (GS). Biofilms in 96-well plates were quantified by spectrophotometry following crystal violet staining (A) and 24 hour biofilms on HA discs were analyzed using FE-SEM (B). The results demonstrate that PBP1a-deficiency significantly decreased biofilm formation by the deficient mutant (* indicates differences at *P*<0.001 when compared to the wild-type, UA159 under the same conditions). Unlike the robust, evenly distributed biofilms of the wild-type, UA159, biofilms of PBP1a mutant, JB467 were sparse and featured with elongated giant cells. In addition, the biofilms of the PBP1a mutant also possessed significantly bigger and seemingly more eDNA nanofibers (indicated by arrows). FE-SEM micrographs were taken at 20k× magnification.

Unlike the parent strain, PBP1a-deficient JB467 was unable to form any biofilms when grown vertically on glass slides (data not shown). Therefore, 96-well plates were used to grow *S*. *mutans* strains UA159, JB467, and JB467C in biofilm medium with glucose (BMG), sucrose (BMS), and glucose plus sucrose (BMGS), and biofilms accumulated at 24 and 48 hours (Fig 6) were measured using a spectrophotometer following crystal violet staining. As expected, wild-type UA159 developed robust biofilms under all different growth conditions tested, especially when grown in medium containing sucrose, which is known to yield copious adhesive glucans [4]. In contrast, however, the PBP1a-deficient mutant, JB467 failed to grow and accumulate any sufficient biofilms in BMG, BMS, or BMGS in 96-well plates (Fig 6A). The most significant differences between UA159 and JB467 were measured when sucrose was included in the growth medium. As shown by FE-SEM analysis with biofilms grown on HA disks (Fig 6B), drastic differences in both quantity and structure of the biofilms were observed between the wild-type and the PBP1a mutant. The wild-type biofilms appeared multi-layered and evenly distributed, while PBP1a mutant had only sparse cell clusters with little or no accumulation of biofilms (Fig 6B). When compared to the wild-type biofilms, the biofilms of the PBP1a mutant also possessed significantly bigger and seemingly more eDNA nanofibers [2] (Fig 6B). Besides, alterations in cellular morphology of the PBP1a-deficient mutant were also apparent, when compared to wild-type UA159 and the complemented strain JB467C (see below for more details).

### PBP1a deficiency causes morphological alterations

**Fig 7:**
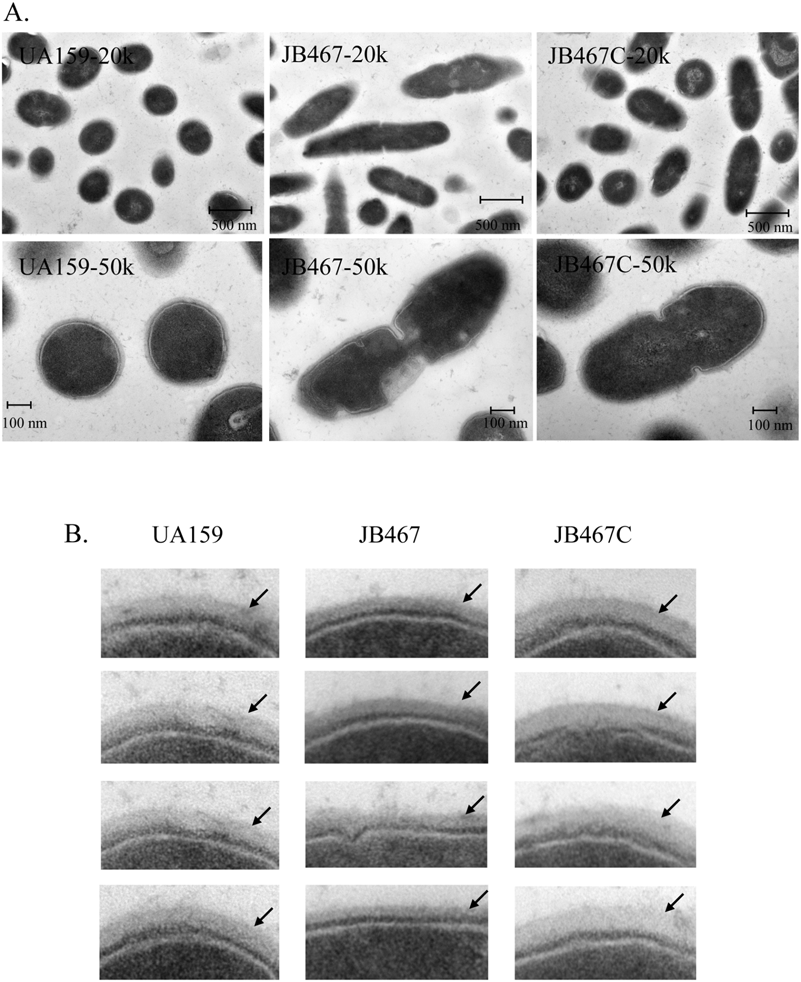
TEM analysis. *S*. *mutans* strains wild-type (UA159), PBP1a-deficient mutant (JB467), and the complement strain (JB467C) were grown in BHI, pH 7.4 to mid-exponential phase (OD_600_nm≈0.3). Panel A shows differences in cell morphology with JB467 displaying elongated cells, defects in cell separation and cells with multiple septa. Images were taken at 20,000x (20k) and 50,000x (50k). Panel B highlights JB467 with a thinner layer of peptidoglycan (indicated by arrows), when compared to the wild-type and the complement strain.

As seen by TEM analysis in Fig 7, PBP1a-deficiency in JB467 caused some major alterations in cellular architecture of the deficient mutant. Unlike the coccoid cells of the wild-type, cells of the PBP1a-deficient mutant were mostly elongated, long rods and unevenly sized, and were characterized with increased interseptal distance, incomplete septum, and presence of the multipartite septa (Fig 7A). Similar morphological features were also observed in biofilms under FE-SEM (Fig 6B). FE-SEM showed that the wild-type was present mostly in short chains of coccoid cells, while the PBP1a mutant displayed mostly in chains of unevenly sized long rods (Fig 6B). In addition, the deficient mutant also appeared to have a significantly thinner PG layer when compared to the parent strain with an average thickness of 21.2±1.12 nm for the mutant vs 28.4±3.2 nm for the wild-type (*P*<0.01) (Fig 7B). Complementation with *pbp1a* in *trans* in JB467C was able to partially restore the phenotypes of JB467 to those similar to the wild-type. The complement strain had an average PG layer of 35.52±4.23 nm.

## Discussion

Like other Gram-positive bacteria, *S*. *mutans* possesses a thick layer of peptidoglycan whose homeostasis is known to be crucial in pathophysiology including cell division and growth, stress tolerance, and biofilm formation. Recent studies have shown that biofilm regulatory protein BrpA, a member of the LytR-CpsA-Psr family of proteins widespread in Gram-positive bacteria, plays a major role in cell envelope homeostasis, influencing cell envelope stress response and biofilm formation [35, 40, 41]. Like BrpA, penicillin-binding protein PBP1a possesses a C-terminal region with rich serine residues that appear to be unique to *S*. *mutans* [27]. Here, we present evidence that PBP1a also plays a critical role in *S*. *mutans* cellular biology and affects its abilities to tolerate acid and oxidative stress and form biofilms, traits critical to the pathogenicity of this bacterium. PBP1a-deficiency causes severe defects in cell division and cell morphology of the mutant, significantly decreases the growth rate and weakens the ability of the deficient mutant to tolerate acid and oxidative stressors, and reduces biofilm formation. However, it is also worthy noting that not all phenotypes of the mutant analyzed were restored fully to the wild-type level by complementation of the deficient mutant with wild-type coding sequence plus its cognate promoter region. Part of the reason could be related to the nature of the complementation system being multiple-copy that is associated with the shuttle vector, pDL278 used (see more details below).

Like *S*. *pneumoniae*, PBP1a-deficiency in *S*. *mutans* is viable when grown in rich medium BHI and semi-defined biofilm medium [29, 34], but significantly affects its growth rate and significantly reduces cell yield in culture OD_600nm_, especially at acidic and alkaline pH environment. The weakened capacity to tolerate low pH, as shown by acid-killing assay, and the compromised abilities to deal with toxic oxygen metabolites such as hydrogen peroxide and superoxide inside and outside the cell, as revealed by paraquat and hydrogen peroxide challenge assays, all are likely contributors to the reduced ability to grow as reflected by the reduced growth rate and culture density.

Unlike other ovococci bacteria, *S*. *mutans* displays heterogeneity among its strains in terms of the length to width ratios, which was shown to be largely due to changes in the K^+^/bicarbonate in the growth medium [42]. Like in *S*. *pneumoniae* and other bacteria [15, 43], our data also further supports the notion that PBP1a in Gram-positive bacteria plays a role in surface-associated cell separation. Unlike the wild-type that mostly exists in chains of ovococci or short rods, the PBP1a-deficient mutant displays mostly in chains of long, mis-shaped rods with incomplete septa and multiple septa. One streptococcal virulence factor that leads to the establishment of biofilms is the innate ability to form chains of bacteria [25]. Likely, the altered cell morphology, especially the long chaining, swelling and aggregation during growth in broth, plays a major role influencing bacterial adherence and biofilm accretion on a surface. PBPs are known to play a crucial role in peptidoglycan synthesis [15, 43]. In *E*. *coli*, PBP1a is shown to be capable of polymerizing lipid II to form a cross-linked peptidoglycan product that contains glycan strands with an average length of ~20 disaccharide units and with ~18–26% of peptides present in cross-links [15]. In the presence of purified peptidoglycan sacculi, PBP1a is capable of attaching a fraction of the newly assembled peptidoglycan to the sacculi via transpeptidase reaction. Consistently, deficiency of PBP1a in *S*. *mutans* leads to development of cells with thinner layer of cell wall.

*S*. *mutans* is known to produce copious extracellular glucose polymers via Gtf enzymes when grown in sucrose rich medium. The Gtf enzymes can also bind to glucans and surfaces of other bacterial species [4, 44], and thus play a central role to *S*. *mutans* dominance in cariogenic plaque and its cariogenicity [4]. When analyzed on 96-well plates, the most drastic difference in biofilm formation between the PBP1a-deificient mutant and the wild-type was measured during growth in the presence of sucrose. As detailed above, PBP1a-deficiency renders major growth defects of the deficient mutant, which is likely the major factor that underlies the reduced biofilm formation by the mutant. It is also possible, but awaits further investigation, however, if the Gtfs’ localization and activity are affected as a result of PBP1a-deficeincy. *S*. *mutans* also releases eDNA, including active releases during growth in biofilms, especially during early biofilms [2, 45] contributing to the extracellular matrices and facilitating bacterial adherence, biofilm formation and structure integrity [2, 46]. Our recently studies have also shown that such release is highly regulated, although the exact mechanism of regulation remains unclear. As shown under FE-SEM, both the wild-type and the PBP1a mutant produce an eDNA network when grown on the HA discs [2]. Relative to the parent strain, the mutant appeared to have more and bigger eDNA nanofibers, although the impact of such eDNA presence and the underlying mechanism remain unclear.

Biofilm formation is a sequential process that is initiated with bacterial adherence to a substratum and followed by formation of microcolonies and accumulation of multilayered cell clusters. During the process, cell envelope plays essential role in not only cell division and multiplication, but also in cell-surface and cell-cell interactions that is known to be crucial in biofilm initiation and development. It is apparent that both chain length and cell shape are aberrant in the PBP1a-deficient mutant. Additionally, defects in remodeling and/or maintenance of the peptidoglycan as a result of PBP1a-deficiency will likely compromise the integrity of the cell envelope and render the cells to be more permeable to protons and other toxic metabolites, which could in part explains the elevated susceptibility to low pH and the superoxide-generating reagent paraquat and hydrogen peroxide. Autolysis, also programmed cell death, is a highly regulated process that can be triggered by stress responses, such as starvation, viral infection and DNA mutations [47]. Unlike the wild-type, the PBP1a mutant displayed a quick and major reduction in OD_600nm_ when incubated in buffered saline with and without Triton X-100, a detergent commonly used in laboratories to permeabilize the membrane of living cells. Such a reduction also suggests that PBP1a mutant may have lost its proper cell envelope protection, a likely result of compromised integrity in response to PBP1a-deficiency. Compromises in cell envelope and cell envelope integrity can be at least in part attributed to the weakened stress tolerance responses observed with the PBP1a-deficient mutant, which in turn influences biofilm formation. In addition, compromises in cell envelope could also influence the localization and function of surface-associated proteins, including the Gtfs, Gbps and SpaP known to play a central role in *S*. *mutans* biofilm formation, directly affect bacterial adherence, inter-cellular interactions and biofilm accumulation, contributing to the reduced biofilms of the PPBP1a-deficient mutant.

Similar to many other Gram-positive bacteria, *S*. *mutans* possesses at least six genes encoding proteins homologous to PBPs, which besides PBP1a (SMU.467), also include one PBP1b (SMU.1991), one PBP2x (SMU.455), one PBP2a (SMU.1949), and two PBP2b (SMU.597 and SMU.889) [27]. It is plausible that the PBP redundancy might suggest that optimal conditions exist in *S*. *mutans* for peptidoglycan biosynthesis and/or homeostasis by different PBPs. As suggested by results of BLAST search, *S*. *mutans pbp1a* gene is most closely linked to genes encoding PBP1a of *Streptococcus agalactiae* (GBS) and other streptococci. In GBS, surface-associated PBP1a, encoded by *ponA*, plays an essential role in resistance of GBS to phagocytosis in a neonatal rat sepsis model, and PonA-deficiency led to an increased susceptibility to antimicrobial peptides [48]. In *S*. *pneumoniae*, alterations of PBP1a are often associated with high level of β-lactam resistance, such as Penicillin G and Cefotaxime [16]. However, it awaits further investigation how deficiency of PBP1a in *S*. *mutans* affects antibiotic resistance. It is also known that major differences exist between the Gram-positive and Gram-negative PBPs, suggesting compensatory mechanisms among the PBPs are likely different. Therefore, it is also possible that overexpression of PBP1a in the complement strain that carries a wild-type *pbp1a* in multiple-copy shuttle vector can cause imbalance between the different PBPs, contributing to the failure to fully restore the phenotypes analyzed to the wild-type. Further studies on the functions of the other PBPs and the interplay of the 6 different PBPs in *S*. *mutans* should provide insights on the underlying mechanisms and other related aspects.

## References

[1] Oli MW, Otoo HN, Crowley PJ, Heim KP, Nascimento MM, Ramsook CB, et al Functional amyloid formation by *Streptococcus mutans* . Microbiol. 2012;158(12):2903–16. 10.1099/mic.0.060855-0 . 23082034 PMC4083658

[2] Liao S, Klein MI, Heim KP, Fan Y, Bitoun JP, Ahn SJ, et al *Streptococcus mutans* extracellular DNA is upregulated during growth in biofilms, actively released via membrane vesicles, and influenced by components of the protein secretion machinery. J Bacteriol. 2014;196(13):2355–66. 10.1128/JB.01493-14 24748612 PMC4054167

[3] Crowley PJ, Brady LJ, Michalek SM, Bleiweis AS. Virulence of a *spaP* mutant of *Streptococcus mutans* in a gnotobiotic rat model. Infect Immun. 1999;67(3):1201–6. . 10024561 10.1128/iai.67.3.1201-1206.1999 PMC96447

[4] Bowen WH, Koo H. Biology of *Streptococcus mutans*-Derived Glucosyltransferases: Role in Extracellular Matrix Formation of Cariogenic Biofilms. Caries Res. 2011;45(1):69–86. 10.1159/000324598 21346355 PMC3068567

[5] Banas JA, Vickerman MM. Glucan-binding proteins of the oral streptococci. Crit Rev Oral Biol Med. 2003;14(2):89–99. . 12764072 10.1177/154411130301400203

[6] Vacca-Smith AM, Bowen WH. Binding properties of streptococcal glucosyltransferases for hydroxyapatite, saliva-coated hydroxyapatite, and bacterial surfaces. Arch Oral Biol. 1998;43(2):103–10. . 9602288 10.1016/s0003-9969(97)00111-8

[7] Navarre WW, Schneewind O. Surface proteins of Gram-positive bacteria and mechanisms of their targeting to the cell wall envelope. Microbiol. Mol. Biol. Rev.: MMBR. 1999;63(1):174–229. 10066836 10.1128/mmbr.63.1.174-229.1999 PMC98962

[8] Silhavy TJ, Kahne D, Walker S. The bacterial cell envelope. Cold Spring Harbor perspectives in biology. 2010;2(5):a000414 10.1101/cshperspect.a000414 20452953 PMC2857177

[9] Banzhaf M, van den Berg van Saparoea B, Terrak M, Fraipont C, Egan A, Philippe J, et al Cooperativity of peptidoglycan synthases active in bacterial cell elongation. Mol Microbiol. 2012;85(1):179–94. 10.1111/j.1365-2958.2012.08103.x . 22606933

[10] Grebe T, Hakenbeck R. Penicillin-binding proteins 2b and 2x of *Streptococcus pneumoniae* are primary resistance determinants for different classes of beta-lactam antibiotics. Antimicrob Agents Chemother. 1996;40(4):829–34. 8849235 10.1128/aac.40.4.829 PMC163214

[11] Martin C, Briese T, Hakenbeck R. Nucleotide sequences of genes encoding penicillin-binding proteins from *Streptococcus pneumoniae* and *Streptococcus oralis* with high homology to *Escherichia coli* penicillin-binding proteins 1a and 1b. J Bacteriol. 1992;174(13):4517–23. 1624444 10.1128/jb.174.13.4517-4523.1992 PMC206242

[12] Martin C, Sibold C, Hakenbeck R. Relatedness of penicillin-binding protein 1a genes from different clones of penicillin-resistant *Streptococcus pneumoniae* isolated in South Africa and Spain. EMBO J. 1992;11(11):3831–6. 1396576 10.1002/j.1460-2075.1992.tb05475.x PMC556892

[13] Reichmann P, Konig A, Linares J, Alcaide F, Tenover FC, McDougal L, et al A global gene pool for high-level cephalosporin resistance in commensal Streptococcus species and *Streptococcus pneumoniae* . J Infect Dis. 1997;176(4):1001–12. . 9333159 10.1086/516532

[14] Dowson CG, Hutchison A, Brannigan JA, George RC, Hansman D, Linares J, et al Horizontal transfer of penicillin-binding protein genes in penicillin-resistant clinical isolates of *Streptococcus pneumoniae* . Proc Natl Acad Sci U S A. 1989;86(22):8842–6. 2813426 10.1073/pnas.86.22.8842 PMC298386

[15] Egan AJ, Vollmer W. The physiology of bacterial cell division. Annals of the New York Academy of Sciences. 2013;1277:8–28. 10.1111/j.1749-6632.2012.06818.x . 23215820

[16] Job V, Carapito R, Vernet T, Dessen A, Zapun A. Common alterations in PBP1a from resistant *Streptococcus pneumoniae* decrease its reactivity toward beta-lactams: structural insights. J Biol Chem. 2008;283(8):4886–94. 10.1074/jbc.M706181200 . 18055459

[17] Sauvage E, Kerff F, Terrak M, Ayala JA, Charlier P. The penicillin-binding proteins: structure and role in peptidoglycan biosynthesis. FEMS Microbiol Rev. 2008;32(2):234–58. 10.1111/j.1574-6976.2008.00105.x . 18266856

[18] Vollmer W, Joris B, Charlier P, Foster S. Bacterial peptidoglycan (murein) hydrolases. FEMS Microbiol Rev. 2008;32(2):259–86. 10.1111/j.1574-6976.2007.00099.x . 18266855

[19] Priyadarshini R, Popham DL, Young KD. Daughter cell separation by penicillin-binding proteins and peptidoglycan amidases in *Escherichia coli* . J Bacteriol. 2006;188(15):5345–55. 10.1128/JB.00476-06 16855223 PMC1540038

[20] Fani F, Leprohon P, Zhanel GG, Bergeron MG, Ouellette M. Genomic analyses of DNA transformation and penicillin resistance in *Streptococcus pneumoniae clinical isolates* . Antimicrob Agents Chemoth. 2014;58(3):1397–403. 10.1128/AAC.01311-13 24342643 PMC3957846

[21] Hughes HV, Lisher JP, Hardy GG, Kysela DT, Arnold RJ, Giedroc DP, et al Co-ordinate synthesis and protein localization in a bacterial organelle by the action of a penicillin-binding-protein. Mol Microbiol. 2013;90(6):1162–77. 10.1111/mmi.12422 24118129 PMC3864544

[22] Vijayan S, Mallick S, Dutta M, Narayani M, Ghosh AS. PBP deletion mutants of *Escherichia coli* exhibit irregular distribution of MreB at the deformed zones. Current Microbiol. 2014;68(2):174–9. 10.1007/s00284-013-0453-z . 24057063

[23] Otero LH, Rojas-Altuve A, Llarrull LI, Carrasco-Lopez C, Kumarasiri M, Lastochkin E, et al How allosteric control of *Staphylococcus aureus* penicillin binding protein 2a enables methicillin resistance and physiological function. Proc Natl Acad Sci U S A. 2013;110(42):16808–13. 10.1073/pnas.1300118110 24085846 PMC3800995

[24] Evans KL, Kannan S, Li G, de Pedro MA, Young KD. Eliminating a set of four penicillin binding proteins triggers the Rcs phosphorelay and Cpx stress responses in *Escherichia coli* . J Bacteriol. 2013;195(19):4415–24. 10.1128/JB.00596-13 23893115 PMC3807471

[25] Berg KH, Stamsas GA, Straume D, Havarstein LS. Effects of low PBP2b levels on cell morphology and peptidoglycan composition in *Streptococcus pneumoniae* R6. J Bacteriol. 2013;195(19):4342–54. 10.1128/JB.00184-13 23873916 PMC3807472

[26] Nguyen UT, Harvey H, Hogan AJ, Afonso AC, Wright GD, Burrows LL. Role of PBPD1 in Stimulation of *Listeria monocytogenes* Biofilm Formation by Subminimal Inhibitory beta-Lactam Concentrations. Antimicrob Agents Chemoth. 2014;58(11):6508–17. 10.1128/AAC.03671-14 . 25136010 PMC4249420

[27] Ajdic D, McShan WM, McLaughlin RE, Savic G, Chang J, Carson MB, et al Genome sequence of *Streptococcus mutans* UA159, a cariogenic dental pathogen. Proc Natl Acad Sci U S A. 2002;99(22):14434–9. . 12397186 10.1073/pnas.172501299 PMC137901

[28] LeBanc D, Lee L. Replication function of pVA380-1 In: Dunny G, Cleary PP, Mckay LL, editors. Genetics and Molecular Biology of Streptococci, Lactococci, and Enterococci. Washington, DC.: ASM Press; 1991 p. 235–9.

[29] Bitoun JP, Nguyen AH, Fan Y, Burne RA, Wen ZT. Transcriptional repressor Rex is involved in regulation of oxidative stress response and biofilm formation by *Streptococcus mutans* . FEMS Microbiol Lett. 2011;320(2):110–7. Epub 2011/04/28. 10.1111/j.1574-6968.2011.02293.x 21521360 PMC3115380

[30] Lau PC, Sung CK, Lee JH, Morrison DA, Cvitkovitch DG. PCR ligation mutagenesis in transformable streptococci: application and efficiency. J Microbiol Methods. 2002;49(2):193–205. Epub 2002/02/07. S0167701201003694 [pii]. . 11830305 10.1016/s0167-7012(01)00369-4

[31] Zeng L, Wen ZT, Burne RA. A novel signal transduction system and feedback loop regulate fructan hydrolase gene expression in *Streptococcus mutans* . Mol Microbiol. 2006;62(1):187–200. . 16987177 10.1111/j.1365-2958.2006.05359.x

[32] Li YH, Lau PC, Lee JH, Ellen RP, Cvitkovitch DG. Natural genetic transformation of *Streptococcus mutans* growing in biofilms. J Bacteriol. 2001;183(3):897–908. 11208787 10.1128/JB.183.3.897-908.2001 PMC94956

[33] Li YH, Lau PC, Tang N, Svensater G, Ellen RP, Cvitkovitch DG. Novel Two-Component Regulatory System Involved in Biofilm Formation and Acid Resistance in *Streptococcus mutans* . J Bacteriol. 2002;184(22):6333–42. . 12399503 10.1128/JB.184.22.6333-6342.2002 PMC151940

[34] Loo CY, Corliss DA, Ganeshkumar N. *Streptococcus gordonii* biofilm formation: identification of genes that code for biofilm phenotypes. J Bacteriol. 2000;182(5):1374–82. 10671461 10.1128/jb.182.5.1374-1382.2000 PMC94426

[35] Wen ZT, Burne RA. Functional genomics approach to identifying genes required for biofilm development by *Streptococcus mutans* . Appl Environ Microbiol. 2002;68(3):1196–203. . 11872468 10.1128/AEM.68.3.1196-1203.2002 PMC123778

[36] Koo H, Hayacibara MF, Schobel BD, Cury JA, Rosalen PL, Park YK, et al Inhibition of *Streptococcus mutans* biofilm accumulation and polysaccharide production by apigenin and tt-farnesol. J Antimicrob Chemoth 2003;52(5):782–9. 10.1093/jac/dkg449 . 14563892

[37] Wen ZT, Burne RA. LuxS-mediated signaling in *Streptococcus mutans* is involved in regulation of acid and oxidative stress tolerance and biofilm formation. J Bacteriol. 2004;186(9):2682–91. . 15090509 10.1128/JB.186.9.2682-2691.2004 PMC387784

[38] Belli WA, Marquis RE. Adaptation of *Streptococcus mutans* and *Enterococcus hirae* to acid stress in continuous culture. Appl Environ Microbiol. 1991;57(4):1134–8. . 1829347 10.1128/aem.57.4.1134-1138.1991 PMC182857

[39] Lemos JA, Chen YY, Burne RA. Genetic and physiologic analysis of the *groE* operon and role of the HrcA repressor in stress gene regulation and acid tolerance in *Streptococcus mutans* . J Bacteriol. 2001;183(20):6074–84. . 11567008 10.1128/JB.183.20.6074-6084.2001 PMC99687

[40] Wen ZT, Baker HV, Burne RA. Influence of BrpA on critical virulence attributes of *Streptococcus mutans* . J Bacteriol. 2006;188(8):2983–92. . 16585759 10.1128/JB.188.8.2983-2992.2006 PMC1447002

[41] Bitoun JP, Liao S, Yao X, Ahn S-J, Isoda R, Nguyen AH, et al BrpA is Involved in Regulation of Cell Envelope Stress Responses in *Streptococcus mutans* . Appl Environ Microbiol. 2012;78(8):2914–22. 10.1128/AEM.07823-11 22327589 PMC3318800

[42] Tao L, MacAlister TJ, Tanzer JM. Factors influencing cell shape in the mutans group of streptococci. J Bacteriol. 1988;170(8):3752–5. 3403512 10.1128/jb.170.8.3752-3755.1988 PMC211357

[43] Massidda O, Novakova L, Vollmer W. From models to pathogens: how much have we learned about *Streptococcus pneumoniae* cell division? Environ Microbiol 2013;15(12):3133–57. 10.1111/1462-2920.12189 . 23848140

[44] Gregoire S, Xiao J, Silva BB, Gonzalez I, Agidi PS, Klein MI, et al Role of Glucosyltransferase B in Interactions of Candida albicans with *Streptococcus mutans* and with an Experimental Pellicle on Hydroxyapatite Surfaces. Appl Environ Microbiol 2011;77(18):6357–67. Epub 2011/08/02. AEM.05203-11 [pii] 10.1128/AEM.05203-11 . 21803906 PMC3187131

[45] Perry JA, Cvitkovitch DG, Levesque CM. Cell death in *Streptococcus mutans* biofilms: a link between CSP and extracellular DNA. FEMS Microbiol Lett 2009;299(2):261–6. Epub 2009/09/09. FML1758 [pii] 10.1111/j.1574-6968.2009.01758.x 19735463 PMC2771664

[46] Hu W, Li L, Sharma S, Wang J, McHardy I, Lux R, et al DNA builds and strengthens the extracellular matrix in *Myxococcus xanthus* biofilms by interacting with exopolysaccharides. PLoS One. 2012;7(12):e51905 10.1371/journal.pone.0051905 PMC3530553 23300576

[47] Koksharova OA. Bacteria and phenoptosis. Biochem Biokhimiia. 2013;78(9):963–70. 10.1134/S0006297913090010 . 24228917

[48] Hamilton A, Popham DL, Carl DJ, Lauth X, Nizet V, Jones AL. Penicillin-binding protein 1a promotes resistance of group B streptococcus to antimicrobial peptides. Infect Immun. 2006;74(11):6179–87. Epub 2006/10/24. 74/11/6179 [pii] 10.1128/IAI.00895-06 . 17057092 PMC1695509

